# Interplay between uncertainty intolerance, emotion regulation, cognitive flexibility, and psychopathology during the COVID-19 pandemic: a multi-wave study

**DOI:** 10.1038/s41598-023-36211-3

**Published:** 2023-06-17

**Authors:** Malvika Godara, Jonas Everaert, Alvaro Sanchez-Lopez, Jutta Joormann, Rudi De Raedt

**Affiliations:** 1 Department of Experimental, Clinical & Health Psychology, Ghent University, Henri Dunantlaan 2, 9000 Ghent, Belgium; 2 Department of Medical and Clinical Psychology, Tilburg University, Tilburg, The Netherlands; 3 Research Group of Quantitative Psychology and Individual Differences, KU Leuven, Leuven, Belgium; 4 Department of Clinical Psychology, Complutense University of Madrid, Madrid, Spain; 5 Department of Psychology, Yale University, New Haven, USA; 6 Present Address: Social Neuroscience Lab, Max Planck Society, Berlin, Germany

**Keywords:** Psychology, Human behaviour

## Abstract

The COVID-19 pandemic has created a significant mental health burden on the global population. Studies during the pandemic have shown that risk factors such as intolerance of uncertainty and maladaptive emotion regulation are associated with increased psychopathology. Meanwhile, protective factors such as cognitive control and cognitive flexibility have been shown to protect mental health during the pandemic. However, the potential pathways through which these risk and protective factors function to impact mental health during the pandemic remain unclear. In the present multi-wave study, 304 individuals (18 years or older, 191 Males), residing in the USA during data collection, completed weekly online assessments of validated questionnaires across a period of five weeks (27th March 2020–1st May 2020). Mediation analyses revealed that longitudinal changes in emotion regulation difficulties mediated the effect of increases in intolerance of uncertainty on increases in stress, depression, and anxiety during the COVID-19 pandemic. Further, individual differences in cognitive control and flexibility moderated the relationship between intolerance of uncertainty and emotion regulation difficulties. While intolerance of uncertainty and emotion regulation difficulties emerged as risk factors for mental health, cognitive control and flexibility seems to protect against the negative effects of the pandemic and promote stress resilience. Interventions aimed at enhancing cognitive control and flexibility might promote the protection of mental health in similar global crises in the future.

## Introduction

The COVID-19 pandemic markedly affected mental health since 2020. The recent report of the Global Burden of Disease study revealed that in the first year of the pandemic alone, the worldwide prevalence of depression increased by an estimated 27.6% while anxiety increased by 25.6%^1^. These worrying trends have been corroborated by longitudinal studies examining the rise in mental health difficulties during the pandemic compared to the pre-pandemic levels^2,3^. A multitude of studies has documented increased levels of depression, anxiety, stress, psychological distress, and poor mental well-being during the pandemic^4–6^. Several studies have shown that certain groups were particularly vulnerable to developing mental health difficulties during the pandemic, such as system-relevant workers^7,8^, children and adolescents^9^, youth populations^10^, women^11,12^, caregivers^13^, and those affected by the COVID-19 disease^14^. Moreover, empirical studies have shown that various previously-identified psychological risk factors for psychopathology also predicted poor mental health outcomes during the pandemic, such as loneliness^15^, negative cognitive biases^16^, worry^17^, ineffective use of coping strategies^18^, low levels of social support^19^, and psychological inflexibility^20^.

Despite the progress that has been made in understanding risk factors that aggravate the impact of the COVID-19 pandemic, there is much that remains to be understood about the interplay between key risk and protective factors for mental health. A recent conceptual framework proposed that the pandemic has challenged people in at least three ways, namely dealing with uncertainty, regulating negative emotions, and flexibly adapting to changing contexts^21^. Difficulties in these domains may engender mental health issues^22–24^. Although only a handful of studies have documented the isolated effects of these aspects on mental health during the pandemic, an integrated approach to understanding their interplay remains absent.

An important vulnerability factor that precipitates mental health difficulties, and that may be of particular relevance during the unpredictable course of a pandemic, is intolerance of uncertainty (IU). IU is conceptualized as negative reactivity to uncertainty or the subjective experience of negative emotionality related to the unknown or uncertain aspects of a given situation, also in the COVID-19 pandemic context^25,26^. Individuals reporting higher levels of IU often perceive, interpret, experience, and respond to uncertainty in a negative manner^27^, leading also to the emergence of dysfunctional behaviors in uncertain situations through maladaptive coping styles^28^. Accordingly, IU has been posited to be a transdiagnostic risk factor for psychopathology and vulnerability^29–34^, and treatment-related reductions in IU have been linked to significantly reduced repetitive negative thinking patterns and is associated with symptom relief in social and generalized anxiety disorders^35^. Given the levels of uncertainty associated with the COVID-19 pandemic due to unexpected lockdowns and a rise in infections, IU is likely to play a key role in precipitating psychopathology in this type of context. Several empirical studies, thus, have confirmed the role of IU in exacerbating psychopathological symptoms^36–38^, psychological distress^39^, insomnia complaints^40^, and loneliness^41^ during the pandemic.

A concept linked with IU is emotion regulation (ER) difficulties, such that recent work has indicated that IU might be inhibiting adaptive ER while increasing difficulties in ER such as the ability to access the repertoire of ER strategies, engendering psychological distress or anxiety^23,42^. Broadly speaking, ER capacities refer to an individual’s ability to understand and manage emotional responses^43^. ER difficulties have been conceptualized as multi-dimensional deficits in emotional awareness and clarity, inability to engage in goal-directed behavior, and inability to successfully apply ER strategies in the face of stress^43^. In general, ER difficulties are associated with increased negative affect and mental health difficulties^44–46^, and reports of increased ER difficulties have also been witnessed during the pandemic^18,47–49^. Conceptual models have linked ER difficulties, especially access to ER strategies, to IU and psychopathology^23,50^, proposing a mediating role of ER difficulties in the relationship between IU and psychopathology. Only a rather small number of studies have empirically investigated the association between IU and ER difficulties^51,52^, including a recent study in the pandemic context that showed maladaptive ER mediated effects of IU on psychological distress^53^. On the other hand, few studies have even suggested an opposing view wherein IU mediates the effect of ER difficulties on psychological distress and negative affect^54,55^. Therefore, further empirical studies, especially in heterogenous community samples, are necessary to investigate the mechanistic pathways linking IU, ER difficulties, and psychological well-being. Specifically, in the COVID-19 pandemic context, increased ER difficulties as a result of higher levels of IU might have thus contributed as an intervening mechanism in the increase in affective psychopathological problems observed during this period.

Contrastingly, cognitive control and flexibility (CCF) has emerged as a protective factor for mental health during the pandemic^20,56,57^. CCF refers to the ability to adapt to dynamic contextual settings and has been linked to individual ability to pursue goal-oriented actions and multi-task or problem solve^58^. Very few studies have investigated the relationship between CCF and psychopathological symptomatology, with one study indicating that CCF was associated with pandemic-related anxiety^59^, and another study outside the pandemic context showing associations with depressive symptom severity^60^. Moreover, a first study examining the relationship between IU, CCF, and psychopathology found evidence for a moderating role of CCF in the relationship between IU and mental health difficulties^61^, indicating that greater levels of CCF supported adaptation to the uncertainty resulting from the COVID-19 pandemic, which further buffered increases in mental health problems. On the other hand, the relationship between CCF and ER difficulties has remained poorly studied as well. Although prior work has extensively shown the association between the various components of cognitive control (updating, set-shifting, and inhibition) and ER difficulties^62^, CCF as a composite reflection of affective control and affective flexibility has not been examined in the context of ER difficulties. In addition to a paucity of cross-sectional studies investigating the protective role of CCF for mental health, this area of investigation has been hampered by a lack of longitudinal studies examining the protective role of CCF in mental health changes during the pandemic and the specific paths through which mental health issues may have increased as a result of individual differences in IU (i.e., increased ER difficulties).

Taking into account how temporal changes in these protective and risk factors for mental health would be especially important within the context of the ever-changing COVID-19 pandemic, we designed a longitudinal study aimed at fully understanding how the pandemic has affected changes in mental health as a function of these factors. Although fragmented lines of research have provided initial evidence for the importance of IU, ER difficulties, and CCF for mental health during the pandemic and outside the pandemic context, there is a dearth of cross-sectional or longitudinal empirical work examining the interplay between these factors in an integrative manner, and consequently, it remains unclear how these factors could be related over time. Addressing this gap, this study also aims to provide a first insight into how these factors interact with each other over time to predict the course of affective psychopathology in stressful situations. Although these constructs have been varyingly conceptualized as more trait-like factors, several studies have shown the malleability of uncertainty intolerance, ER capacities, and CCF, indicating their amenability to change as a result of external manipulations due to dynamic contexts^63–66^. This indicates that perhaps as a consequence of an acute external stressor, such as the COVID-19 pandemic context, different patterns of IU, ER difficulties and CCF could have been observed in relation to changes in psychopathology. Thus, in the present study, we used a longitudinal design that comprised a total of 5 weekly assessments of the variables of interest, during the initial months of the COVID-19 pandemic and the associated lockdown in 2020.

In line with recent models of resilience and vulnerability processes in the pandemic context^21^, IU is expected to predict psychopathology, and ER difficulties and CCF are considered to modulate the impact of this factor on the trajectory of psychopathology. In line with these theoretical models, studies have vouched for a mediating effect of ER difficulties in the relationship between IU and psychopathology^23,67^, and another study has shown that different levels of CCF moderate the relation between IU and psychopathology^61^. As such, we had two main aims in the present study. First, we aimed to examine whether the trajectory of IU predicts the development of psychopathological (i.e., depression, anxiety, stress) symptoms longitudinally and whether this relationship is mediated by experienced ER difficulties. Second, we aimed to test whether CCF plays a protective moderating role in the relationship between IU and ER difficulties. Accordingly, we hypothesized that increases in IU will predict increases in depression, anxiety, and stress, and this relationship will be mediated by an increase in ER difficulties over time. Moreover, we explored whether changes in CCF over time will moderate the relationship between increases in IU and increases in ER difficulties.

## Method

### Participants

A total of 317 individuals took part in the study, out of which 13 individuals were excluded due to data quality reasons (see below). Therefore, the final sample comprised 304 individuals (Mean age (SD) = 34.97 (12.02) years, Mode of age group = 25–34 years, age range = 18 years or older, 191 Males), who were residents of the United States of America at the time the study was conducted. Table 1 presents sample characteristics. Participants were recruited using the online platform Amazon’s Mechanical Turk (MTurk), which provides access to large and diverse samples that are suitable for psychological research^68^. The participants were recruited from MTurk using CloudResearch, which is a participant-sourcing platform for online studies. We recruited participants from MTurk who had a history of providing high-quality responses, i.e., they had an acceptance ratio of ≥ 95% in other online studies they participated in, and who were located in the USA. Before participation, all individuals provided informed consent and were compensated for their participation at $10 per hour. The study was approved by the Institutional Review Board of Yale University and all study procedures were carried out in accordance with the Declaration of Helsinki.

**Table 1:** An overview of the sample characteristics for the final sample N = 304.

Demographics	N	%
Sex		
Male	191	62.8
Female	112	36.8
Other	1	0.4
Age group		
18–24 years	39	12.8
25–34 years	155	50.9
35–44 years	53	17.4
45–54 years	36	11.8
55–64 years	15	4.9
Above 64 years	6	2.2
Highest education level		
Less than high school	0	0
High school	24	7.8
Some college	45	14.8
2-year degree	26	8.5
Undergraduate degree	146	48.02
Graduate degree	63	20.8
Ethnic category		
American Indian or Alaska Native	3	0.9
Asian	20	6.5
Black or African American	49	16.1
Hispanic or Latino	19	6.2
Native Hawaiian or Pacific islander	2	0.6
White	211	69.7
Employment status		
Employed full time	197	64.8
Employed part-time	33	10.8
Unemployed & currently looking for work	19	6.2
Unemployed & currently not looking for work	7	2.3
Student	9	2.9
Retired	8	2.6
Homemaker	5	1.6
Self-employed	24	7.8
Unable to work	2	1
Psychiatric medicine		
No	272	89.4
Yes	32	10.6
Familiarity with public health and safety guidelines (at baseline)		
Not at all familiar	0	0
Somewhat familiar	5	1.74
Neutral	26	8.5
Very familiar	136	44.7
Completely familiar	137	45.06
**“**Have you or someone in your nearest environment (friends, family, co-workers) tested positive for or have possibly contracted coronavirus or COVID-19?” (at baseline)		
Yes/maybe	95	31.3
No	209	68.7

### Measures

Participants were administered a battery of questionnaires in the project. The following questionnaires were administered at 5 measurement occasions: Intolerance of Uncertainty Scale-12 (IUS), Depression, Anxiety and Stress Scale-21 (DASS-21), Cognitive Control and Flexibility Questionnaire (CCFQ), Difficulties in Emotion Regulation Scale-Short Form (DERS), Cognitive Emotion Regulation Questionnaire (CERQ), Perseverative Thinking Questionnaire (PTQ), Future-oriented Repetitive Thinking scale (FoRT), Perceived Stress Scale (PSS), and Brief Resilience Scale (BRS). The Connor-Davidson Resilience Scale (CD-RISC 10) was administered only at the first timepoint. Participants completed the questionnaires at five timepoints over a span of five weeks, with one-week time intervals. However, given the research questions of interest in the current study, the following measures are discussed:

#### Intolerance of uncertainty

Participants’ IU was assessed using the IUS-12^69^. Participants rated 12 items (e.g., “It frustrates me not having all the information I need”) on a 5-point Likert scale ranging from 1 (not at all characteristic of me) to 5 (entirely characteristic of me). Higher scores on the scale indicate higher levels of IU. The internal consistency (Cronbach’s alpha) for IUS-12 hovers around 0.93 for heterogenous samples^70^ (comprising clinical and non-clinical populations), and accordingly Cronbach’s alpha for IU across the five measurement occasions in the current study was as follows: 0.91, 0.92, 0.93, 0.92, and 0.92.

#### Psychopathology

DASS-21^71^ was used to assess levels of depression (e.g., “I felt that I had nothing to look forward to”), anxiety (e.g., “I felt I was close to panic”), and stress (e.g., “I found it difficult to relax”). Participants rated 21 items on 3 sub-scales, each containing 7 questions, using a 4-point Likert scale ranging from 0 (“Does not apply to me at all”) to 3 (“Applies to me completely”). Higher scores on the sub-scales indicate higher levels of depression, anxiety, and stress. Internal consistency for depression subscale ranges between 0.88 and 0.94, for anxiety subscale between 0.80 and 0.87, and for stress subscale between 0.84 and 0.91^72^. In the present study, Cronbach’s alpha for the Depression subscale across the five measurement occasions was as follows: 0.92, 0.90, 0.90, 0.88, and 0.88. The Cronbach’s alpha for the Anxiety subscale were as follows: 0.93, 0.92, 0.91, 0.90, and 0.91. The Cronbach’s alpha for the Stress subscale were as follows: 0.91, 0.90, 0.90, 0.89, and 0.88.

#### Emotion regulation difficulties

We assessed ER difficulties using the 18-item DERS-Short Form^73^. We assessed three domains of ER abilities proposed by conceptual models^43^: understanding of emotions, ability to act in accordance with goals when experiencing negativity, and ability to use situationally appropriate ER strategies. DERS consists of two subscales assessing the domain ‘understanding of emotions’: ‘Lack of emotional clarity’ and ‘Lack of emotional awareness’. The domain ‘ability to act in accordance to goals when experiencing negativity’ is reflected in two subscales in DERS: ‘Difficulties engaging in goal-directed behavior’ and ‘Impulse control difficulties’. We assessed only one of the subscales for both these domains to avoid participants’ fatigue due to completing lengthy surveys at each timepoint. Meanwhile, the domain ‘ability to use situationally appropriate emotion regulation strategies’ has only one subscale in DERS. Therefore, we assessed three subscales ‘Lack of emotional clarity’ (e.g., “I have difficulty making sense out of my feelings”), ‘Difficulties engaging in goal-directed behavior’ (e.g., “When I’m upset, I have difficulty getting work done”), and ‘Limited access to effective emotion regulation strategies’ (e.g., “When I'm upset, I believe that wallowing in it is all I can do”) that are summed up to form a global indicator of ER difficulties at each wave. Participants rated 9 items on a 5-point Likert scale ranging from 1(“Almost never”) to 5 (“Almost always”). Higher scores indicate greater ER difficulties. In the original study^73^, the internal consistency for DERS-short form was 0.91 for the combined scale, and in the present study, the Cronbach’s alpha for ER difficulties across the five measurement occasions was 0.95 for each timepoint.

#### Cognitive control and flexibility

Participants completed the 18-item CCFQ^58^. The scale consists of 2 sub-scales, ‘cognitive control over emotions’ (e.g., “My thoughts and emotions interfere with my ability to concentrate”) and ‘coping and appraisal flexibility’ (e.g., “I approach the situation from multiple angles”) that are summed up to form a global indicator of CCF at each wave. Participants provided ratings using a 7-point Likert scale ranging from 1 (“Strongly disagree”) to 7 (“Strongly agree”). Higher scores indicate higher levels of cognitive control over emotion and higher levels of coping and appraisal flexibility. In the original study^58^, internal consistency for CCFQ ranged between 0.89 and 0.93 for separate subscales in community and student samples, and in the present study, the Cronbach’s alpha for the CCF scores across the five measurement occasions were as follows: 0.87, 0.91, 0.92, 0.93 and 0.93.

### Procedure

Participants were administered the questionnaires using a Qualtrics online survey tool over five measurement occasions (27th March 2020–1st May 2020), once a week. During the period of data collection, the majority of the states in the USA were under stay-at-home orders^74^. Five states did not issue any stay-at-home orders. Two participants from the present sample were located in states with no stay-at-home orders (Iowa and North Dakota). At baseline (first measurement occasion), prior to the questionnaires of interest, participants first completed the informed consent. Then participants provided information regarding the following demographic variables: age, sex, highest education level, ethnic category, employment status, presence of a current psychiatric diagnosis, whether they or someone in their environment had a positive COVID-19 test, and their overall familiarity with pandemic-related health guidelines. Next, participants completed the independent and dependent measures of interest. The questionnaires were administered in a randomized order. During the surveys, two reading check questions (two Captcha verification items) were presented to ensure that participants were paying attention to the survey, and participants were required to answer both items correctly. Further, seven reading check items (“To indicate you are reading, select 0 for this item”) were intermixed with the various questionnaire items to assess whether participants were reading the items carefully. These reading check items were presented at irregular intervals and participants were required to correctly answer all of them. Data from participants failing to correctly answer these quality control questions were excluded from further analysis (n = 13). At the subsequent four measurement occasions, reading check items were not presented. Participants had to complete at least 3 waves of data collection (including baseline mandatorily) to be included in the analyses.

### Statistical approach

As a first step in our main analysis strategy, we evaluated the missingness patterns in our dataset. Although we had no missing data at baseline due to the nature of our data collection (participants obligated to complete the baseline measure to participate in the rest of the weekly assessments), we saw some longitudinal dropouts in our data. As such, we had the following percentage of missing data due to this longitudinal dropout at measurement occasions 2, 3, 4, and 5 respectively: 31.46%, 35.80%, 37.67%, and 44.68% of the initial sample who completed timepoint 1. We then performed multiple imputation procedure for the missing data for these four measurement occasions using the predictive mean matching approach. As recommended, we included into the imputation model all variables that were either predictors of missingness or predictors of the value of the incomplete variable for multiple imputation^75^. We imputed all missing data using multiple imputation by chained equations using the mice package in R^76^. Pooling of results was done using Rubin's rule for multiple imputation inference^77^.

**Figure 1:**
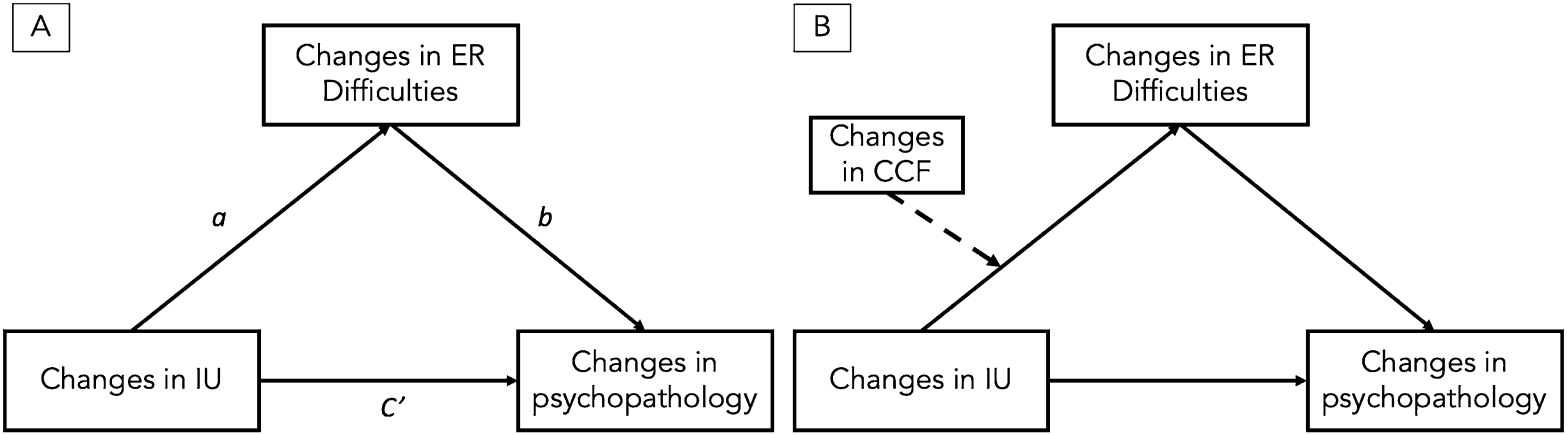
(**A**) The mediation model. (**B**) The moderated mediation model. *IU* Intolerance of Uncertainty, *ER difficulties* Emotion Regulation difficulties, *CCF* Cognitive Control and Flexibility, and psychopathology refers to depression, anxiety, and stress.

For all our variables, we first ran growth models using lavaan over imputed datasets and pooled fit indices were obtained using lavaan and semTools packages^78,79^. The hypothesized mediation model and moderated mediation model (see Fig. 1A,B) were then tested in two separate models each for depression, anxiety, and stress using a bootstrapping approach^80^. Lavaan package for R was used to run the hypothesized models with bias-corrected 95% confidence intervals (n = 5000) to test the significance of the indirect (i.e., mediated) effects (i.e., IU → ER difficulties → psychopathological outcomes: depression, anxiety, stress) moderated by the slope of CCF, i.e., conditional indirect effects.

#### Preliminary growth modeling of variables of interest

First, separate normative growth trajectories were constructed using data at the five timepoints for IU, ER difficulties, CCF, depression, anxiety and stress. In each of the growth models, the factor loadings for the five timepoints were fixed to 1 on the intercept factor to signify that the intercepts are invariant across time. Since it was expected that there would be a linear increase in growth of the variables over time, the factor loadings relating the five measures of the slope factors were fixed to linear slope. The latent intercept and latent slope factors were estimated for the variables. Model fit was assessed using three fit indices: root-mean-square error of approximation (RMSEA), comparative fit index (CFI), and Tucker–Lewis index (TLI). RMSEA smaller than 0.06 and a CFI and TLI larger than 0.95 indicate a relatively good model–data fit in general. The growth models for each of the variables were run over multiple imputed dataset and pooled fit indices were derived. Pooled fit indices are reported for growth models that converged across 30 imputed datasets.

#### Mediation and moderated mediation analyses

To construct the mediation and moderated mediation models to test our hypotheses concerning the impact of changes in the predictor, the mediator, and the moderator on changes in the psychopathology outcomes over time, we extracted latent slope scores from the growth models. These latent slope scores from five random imputations were then used to test our mediation and moderated mediation models in the next step. In both the mediation and moderated mediation models, the linear slope of IU was the predictor variable and the linear slope of ER difficulties was the mediator. In the moderated mediation model, the slope of CCF was added as the moderator of the relationship between the slopes of IU and ER difficulties. The three outcome variables were the linear slopes of depression, anxiety, and stress. Moderated mediation analyses test the conditional indirect effect of a moderating variable (i.e., changes in CCF) on the relationship between a predictor (i.e., changes in IU) and an outcome variable (i.e., changes in psychopathology) via potential mediators (i.e., changes in ER difficulties). This moderated mediation model explicitly tests the moderating effect on the predictor to mediator path (i.e., path a). An index of moderated mediation was used to test the significance of the moderated mediation, i.e., the difference in the indirect effects across different levels of changes in CCF. Significant effects were supported by the absence of zero within the confidence intervals. All estimates and bootstrapped standard errors provided for mediation and moderated mediation models are pooled across five imputations. Age and sex were not found to be significantly associated with IU, ER difficulties, CCF, depression, anxiety, and stress in the present data. Therefore, they were not included as covariates in the present mediation and moderated mediation analyses.

#### Testing the direction of effects

To determine the direction of effects for the predictor and mediator on mental health outcomes, we conducted follow-up cross-lagged mediation analyses. We ran separate models with depression, anxiety, and stress as outcomes, and tested the effects of IU and ER difficulties as predictor or mediator, pooling results over 20 imputed datasets. IU (predictor) at timepoint 1, 2 and 3 predicted ER difficulties (mediator) at timepoints 2, 3 and 4 respectively, and the opposing relation with ER difficulties as predictor and IU as mediator was also modeled. ER difficulties at measurement occasions 2, 3 and 4 then predicted psychopathology (outcome) at measurement occasions 3, 4 and 5 respectively, and the same relation was also tested with IU as mediator. Pooled estimates, standard errors and p-values are reported.

## Results

### Preliminary growth modeling of variables of interest

Separate linear growth models were estimated and found to fit well for IU (pooled CFI = 0.96, TLI = 0.96, and RMSEA < 0.001 [0.000–0.023]), ER difficulties (pooled CFI = 1.00, TLI = 1.01, and RMSEA = 0.045 [0.000–0.084]), and CCF (pooled CFI = 1.00, TLI = 1.07, and RMSEA < 0.001 [0.000–0.060]). Overall, across the five timepoints, participants experienced an increase in IU (mean intercept = 35.74, *p* < 0.001; mean slope = 0.92, *p* < 0.001) and ER difficulties (mean intercept = 21.47, *p* < 0.001; mean slope = 0.87, *p* < 0.001), suggesting relative intraindividual variability in IU and ER difficulties across time. On the other hand, participants experienced a decrease in CCF across the timepoints (mean intercept = 70.78, *p* < 0.001; mean slope = − 1.60, *p* = 0.015). There was no significant estimate for the covariance between the intercept and slope factors for IU (estimate = 2.34, *p* = 0.457), ER difficulties (estimate = 2.74, *p* = 0.306), and CCF (estimate = 2.03, *p* = 0.945). This indicates that individuals starting at high levels of IU, ER difficulties or CCF were not necessarily the same people who reported greater increases or decreases in the variables over time.

Similarly for psychopathological outcomes, separate linear growth models were estimated and found to fit the data well, for anxiety (pooled CFI = 1.00, TLI = 1.01, and RMSEA < 0.001 [0.000–0.000]), stress (pooled CFI = 1.00, TLI = 1.03, and RMSEA < 0.001 [0.000–0.000]) and depression (pooled CFI = 1.00, TLI = 1.01, and RMSEA < 0.001 [0.000–0.000]). Overall, across timepoints, participants experienced an increase in anxiety (mean intercept = 6.86, *p* < 0.001; mean slope = 0.23, *p* = 0.023), stress (mean intercept = 7.79, *p* < 0.001; mean slope = 0.20, *p* = 0.033) and depression (mean intercept = 7.71, *p* < 0.001; mean slope = 0.15 *p* = 0.042). This suggests that there was relative intraindividual variability in anxiety, stress and depression across time. There was no significant estimate for the covariance between the intercept and slope factors of anxiety (estimate = 1.04, *p* = 0.184), stress (estimate = 0.41, *p* = 0.559) and depression (estimate = 0.49, *p* = 0.521). This indicates that individuals starting at high levels of psychopathology did not necessarily report greater increases in these variables over time. Supplementary Table S1 provides descriptives for IUS-12, DERS, CCFQ, and three subscales of DASS-21 at each of the five measurement occasions, and the correlations between the slopes of IU, ER difficulties, CCF, depression, anxiety, and stress.

### Mediation and moderated mediation analyses

#### Mediation models

The slope of IU was significantly associated with the slope of ER difficulties in models of depression (estimate = 0.50, boot SE = 0.07), anxiety (estimate = 0.64, boot SE = 0.11) and stress (estimate = 0.59, boot SE = 0.10). This indicates that increases in IU were associated with increases in ER difficulties. Changes in ER difficulties were significantly associated with increases in depression (estimate = 0.09, boot SE = 0.002), anxiety (estimate = 0.07, boot SE = 0.001), and stress (estimate = 0.07, boot SE = 0.0001). Increases in the association between the changes in IU and ER difficulties were associated with increases in depression (pooled estimate ab = 0.03, pooled boot SE = 0.003), anxiety (pooled estimate ab = 0.04, pooled boot SE = 0.001), and stress (pooled estimate ab = 0.04, pooled boot SE = 0.004). Lastly, independent of its association with increases in ER difficulties, there was also a significant direct effect of changes in IU on increases in depression (estimate = 0.13, boot SE = 0.002), anxiety (estimate = 0.40, boot SE = 0.005), and stress (estimate = 0.18, boot SE = 0.002). This indicates a partial mediation effect of ER difficulties on depression, stress and anxiety. All 95% bootstrapped confidence intervals for the effects across the five imputations are reported in Table 2.

**Table 2:** 95% bootstrapped confidence intervals for the different paths of the mediation models for depression, anxiety and stress.

Model	Imputation	95% bias-corrected bootstrapped confidence intervals			
		a	b	c	ab
Depression	1	0.04, 0.55	0.10, 0.17	0.07, 0.20	0.01, 0.08
	2	0.06, 0.66	0.06, 0.12	0.15, 0.33	0.01, 0.07
	3	0.14, 0.94	0.08, 0.15	0.12, 0.32	0.02, 0.11
	4	0.10, 1.38	0.01, 0.02	0.06, 0.10	0.002, 0.02
	5	0.12, 1.08	0.04, 0.08	0.01, 0.11	0.01, 0.07
Anxiety	1	0.06, 0.65	0.03, 0.11	0.42, 0.60	0.01, 0.06
	2	0.12, 0.94	0.03, 0.12	0.55, 0.85	0.01, 0.09
	3	0.09, 1.39	0.03, 0.07	0.12, 0.28	0.08, 0.08
	4	0.11, 1.10	0.05, 0.17	0.24, 0.62	0.01, 0.15
	5	0.17, 1.88	0.02, 0.05	0.12, 0.26	0.01, 0.08
Stress	1	0.04, 0.54	0.04, 0.10	0.11, 0.19	0.003, 0.04
	2	0.06, 0.66	0.06, 0.15	0.27, 0.46	0.01, 0.08
	3	0.10, 1.39	0.01, 0.04	0.003, 0.12	0.003, 0.05
	4	0.10, 1.07	0.04, 0.09	0.07, 0.24	0.01, 0.08
	5	0.16, 1.89	0.04, 0.07	0.05, 0.24	0.01, 0.12

#### Moderated mediation models

In the next step, we added the slope of CCF as the moderator of the relation between the slopes of IU and ER difficulties to the three models of depression, anxiety and stress. The slope of CCF significantly moderated changes in ER difficulties (estimate = 0.69, boot SE = 0.20) such that stronger reductions in CCF were associated with increases in ER difficulties. There was a significant effect of the slope of IU on the slope of ER difficulties (estimate = 2.18, boot SE = 0.88). Importantly, an interaction between the slope of IU and the slope of CCF on the slope of ER difficulties was significant (estimate = 1.01, boot SE = 0.35). Tests of simple slopes (i.e., conditional effects on path *a* at mean and ± 1 SD) found a significant association between the slope of IU and the slope of ER difficulties only for those who had higher levels of reductions in CCF (estimate = 1.46, boot SE = 0.28), but not for those who had low levels of reductions in CCF (estimate = 0.19, boot SE = 0.29). This indicates that increases in IU led to increases in ER difficulties specifically for individuals who showed high levels of reductions in CCF over time. Increases in ER difficulties, in turn, significantly predicted increases in depression (estimate = 0.08, boot SE = 0.001), anxiety (estimate = 0.05, boot SE = 0.0003) and stress (estimate = 0.04, boot SE = 0.0001) over time. The overall moderated mediation model was supported by a significant index of moderated mediation for models of depression (estimate = 0.04, boot SE = 0.001), anxiety (estimate = 0.04, boot SE = 0.001), and stress (estimate = 0.03, boot SE = 0.001). As zero is not within the 95% bootstrapped CI across the five imputations (see Table 3), this indicates a significant moderating effect of reductions in CCF on the indirect effect of increases in IU on increases in depression, anxiety and stress via increases in ER difficulties. The conditional indirect effect was significant in those with higher levels of reductions in CCF in models of depression (estimate = 0.06, boot SE = 0.001), anxiety (estimate = 0.05, boot SE = 0.001), and stress (estimate = 0.05, boot SE = 0.001). However, the conditional indirect effect was significant in those with lesser reductions in CCF in models of depression (estimate = 0.01, boot SE = 0.001), anxiety (estimate = 0.01, boot SE = 0.001), and stress (estimate = 0.004, boot SE = 0.001). This indicates that only in individuals who had reductions in CCF over time, increases in the association between IU and ER difficulties led to increases in depression, anxiety and stress. Lastly, there was significant evidence that independent of its association with increases in ER difficulties, increases in IU were also directly associated with increases in depression (estimate = 0.23, boot SE = 0.003), anxiety (estimate = 0.18, boot SE = 0.004), and stress (estimate = 0.16, boot SE = 0.002). All 95% bootstrapped confidence intervals for the effects across the five imputations are reported in Table 3.

**Table 3:** 95% bootstrapped confidence intervals for the index of moderated mediation and conditional indirect effects of moderator for the moderated mediation models of depression, anxiety and stress.

Model	Imputation	95% bias-corrected bootstrapped confidence intervals		
		Index of moderated mediation	Conditional indirect effects at low levels of moderator	Conditional indirect effects at high levels of moderator
Depression	1	0.01, 0.05	− 0.01, 0.02	0.02, 0.06
	2	0.02, 0.07	0.003, 0.12	0.04, 0.16
	3	0.00004, 0.05	− 0.01, 0.01	0.003, 0.03
	4	0.001, 0.05	− 0.01, 0.02	0.01, 0.04
	5	0.01, 0.11	− 0.04, 0.03	0.03, 0.13
Anxiety	1	0.01, 0.04	0.002, 0.08	0.02, 0.10
	2	0.001, 0.04	− 0.01, 0.02	0.05, 0.002
	3	0.01, 0.15	− 0.04, 0.05	0.02, 0.15
	4	0.01, 0.22	− 0.05, 0.09	0.04, 0.24
	5	0.01, 0.10	− 0.11, 0.03	0.01, 0.05
Stress	1	0.008, 0.38	− 0.002, 0.06	0.02, 0.07
	2	0.001, 0.011	− 0.0002, 0.0001	0.001, 0.02
	3	0.002, 0.09	− 0.02, 0.03	0.01, 0.08
	4	0.01, 0.11	− 0.04, 0.05	0.03, 0.14
	5	0.02, 0.16	− 0.06, 0.07	0.05, 0.22

#### Testing the direction of effects

We found significant indirect effects of IU at measurement occasions 1, 2, and 3 on depression at timepoint 3, 4, and 5 via ER difficulties at timepoints 2 (b = 0.05, se = 0.02, *p* = 0.001), 3 (b = 0.06, se = 0.01, *p* < 0.001), and 4 (b = 0.06, se = 0.01, *p* < 0.001) respectively. Similarly, we also found significant indirect effects of IU at timepoint 1, 2, and 3 on anxiety at timepoint 3, 4, and 5 via ER difficulties at timepoints 2 (b = 0.08, se = 0.03, *p* = 0.005), 3 (b = 0.06, se = 0.01, *p* < 0.001), and 4 (b = 0.03, se = 0.01, *p* = 0.028) respectively. Moreover, we also found significant indirect effects of IU at timepoint 1, 2, and 3 on stress at timepoint 3, 4, and 5 via ER difficulties at timepoints 2 (b = 0.09, se = 0.03, *p* = 0.001), 3 (b = 0.04, se = 0.01, *p* = 0.003), and 4 (b = 0.04, se = 0.01, *p* = 0.002) respectively. This implies that IU at present timepoint predicted ER difficulties at next timepoint which further predicted psychopathology at the future timepoint. However, for the reverse relation, no significant effects were observed for the indirect effect of ER difficulties at timepoints 1, 2, and 3 on depression, at timepoints 3, 4, and 5 via IU at timepoints 2 (b = 0.04, se = 0.02, *p* = 0.052), 3 (b = 0.03, se = 0.03, *p* = 0.42), and 4 (b = 0.01, se = 0.01, *p* = 0.32) respectively. Similarly, no significant effects were observed for the indirect effect of ER difficulties at timepoints 1, 2, and 3 on stress, at timepoints 3, 4, and 5 via IU at timepoints 2 (b = 0.01, se = 0.02, *p* = 0.494), 3 (b = 0.02, se = 0.03, *p* = 0.065), and 4 (b = 0.01, se = 0.01, *p* = 0.551) respectively. Lastly, no significant effects were also observed for the indirect effect of ER difficulties at timepoints 1, 2, and 3 on anxiety, at timepoints 3, 4, and 5 via IU at timepoints 2 (b = 0.02, se = 0.02, *p* = 0.489), 3 (b = 0.004, se = 0.01, *p* = 0.785), and 4 (b = 0.01, se = 0.01, *p* = 0.280) respectively. Meanwhile, IU and ER difficulties at timepoint *t* significantly predicted each other at next timepoint *t* + *1*. While ER difficulties at timepoints *t* and *t* + *1* predicted psychopathology at *t* + *2*, IU at *t* or *t* + *1* did not. These findings provide evidence for the model IU → ER difficulties → Depression/Anxiety/Stress, but not for the reversed mediation model ER difficulties → IU → Depression/Anxiety/Stress. The findings imply that while IU and ER difficulties share a mutually reinforcing relationship, longitudinally IU exerts influence over the development of psychopathology through exacerbation of ER difficulties over time. This suggests that models with IU predicting mental health difficulties via ER difficulties are the most appropriate model in the present study context. These results are in line with models of vulnerability and resilience that have proposed that the impact of IU on psychopathology could be mediated by ER difficulties^23,27^.

## Discussion

The goals of the present study were twofold. First, we aimed to examine whether changes in ER difficulties mediated the effect of IU on mental health difficulties during the COVID-19 pandemic, in a period that was characterized by a particular context of uncertainty (i.e., the lockdown/stay-at-home mandatory or advisory orders from March 1 to May 31, 2020 in USA). Our second aim was to investigate whether individual differences in CCF moderated the relationship between IU and ER difficulties. Overall, our findings suggest that increases in IU over time were associated with increases in the levels of depression, anxiety, and stress experienced across that period. Importantly, this effect was mediated by its association with increases in ER difficulties over time. This implies that individuals who experienced increasing difficulties in tolerating uncertain situations, such as the changing aspects of the pandemic and the related lockdowns, also experienced increased difficulties in regulating their emotional responses, which were further associated with developing a higher level of mental health problems. However, very importantly, this indirect effect was only observed in individuals who had steep reductions in CCF over time, but not for individuals who had little or no changes in CCF levels over the same time.

These results have several relevant implications. First, our longitudinal findings support previous cross-sectional studies that have documented mental health challenges during the COVID-19 pandemic related to IU^36–38,40,41^ and ER difficulties^18,47–49^. However, importantly, we extend those findings by showing that IU during the pandemic was related to mental health directly but also indirectly through its association with ER difficulties over time. Our findings also provide evidence to support the notion that ER difficulties serve as a mediator for the relationship between IU and psychopathology, but the reversed relationship is not supported. The small number of cross-sectional studies that have examined the relationships between IU, ER difficulties, and psychopathology have varyingly shown both IU and ER difficulties as the mediator^51,52,54,55^. However, the findings from the longitudinal cross-lagged models in the present study provide a clear directional effect. These findings are in line with frameworks that have posited that IU leads to negative cognitive and emotional reactions, inhibiting adaptive emotion regulation, which further leads to increased psychopathological symptoms^23,27^. This implies that individuals who are unable to tolerate uncertain situations have difficulties regulating their negative emotional responses incurred due to the situation which leads to them exhibiting higher levels of stress, depression, or anxiety. The COVID-19 pandemic brought with it many uncertainties, ranging from the nature and evolution of the virus and disease, the initial lack of an effective vaccine program, social contact limitations, economic uncertainties and continuously changing public health and social restriction norms. Individuals who were high on IU were unable to successfully regulate the negative emotional responses owing to these uncertain situations of the pandemic.

The swiftly evolving nature of the pandemic necessitated swift adaptation to changing situations, requiring people to regulate their emotional responses in line with the current situation. Accordingly, our findings indicate that higher levels of CCF during the pandemic likely buffered the effects of IU on mental health by moderating its influence on ER difficulties. Our findings are in line with previous studies that have shown that CCF dampens the effects of IU, serving as a protective factor for mental health^56,61,81^. A recent study showed that individuals exhibiting lower levels of cognitive flexibility find it difficult to engage adaptive emotion regulation strategies in stressful situations, which inhibits coping and adaptation to adversity leading to greater levels of psychopathology^58^. Theoretical models have posited CCF capacities to be reflected in executive functioning skills which allow an individual to flexibly adapt to the current contextual demands^24,82^. Within the context of the COVID-19 pandemic, the rapidly changing situations demanded flexible adaptation to evolving restrictions and isolation measures. As such, our findings indicate that individuals who were lower on these flexibility capacities, or worsened on them over time, had difficulties adapting their emotion regulation capacities.

A practical implication of these findings is that individuals experiencing conditions of IU might benefit from receiving training in cognitive control and flexibility to promote better emotion regulation and protect mental health in the face of major stressors^66^. Studies have shown that trainings aimed at enhancing cognitive control and broad flexibility capacities lead to improved emotion regulation capacities, lower levels of psychopathological symptoms and yield resilient adaptation to stressful situations^83–85^. Such scalable and easily applicable interventions might prove especially effective to tackle mental health difficulties in times of global crises. Furthermore, the use of such trainings in combination with present state-of-the-art prevention and treatment programs for affective or anxiety disorders, in an ad-hoc manner, could promote improved outcomes.

Despite the strengths of the study, some important limitations of the present work must be considered. First, an important limitation concerns the investigated period of 5 weeks. The study aimed to look at the trajectories of mental health, its risk and protective factors, but it only covered an initial phase of the lockdown imposed in the USA in 2020. However, certain mental health effects of the pandemic might only become evident over a longer period of time due to cumulative stress over time^86^. Moreover, longer time frames of assessment might also reveal the (in)stability of the associations between IU, ER difficulties and CCF. Regardless, our findings show a clear pattern of immediate effects of the pandemic and the lockdown on mental health, although studies with longer timeframes might be able to further clarify the evolution of these trajectories. Another limitation concerns the self-report nature of our measures. While our measures show good internal consistency across measured timepoints, the questionnaires might fail to capture the cognitive-behavioral components of assessed variables. Therefore, studies examining the effects of IU, flexibility and ER implementation on psychopathology will benefit from the use of more ecologically valid behavioral measures. Furthermore, another limitation of the study concerns the sample of study. Given the nature of online recruitment, we were not able to recruit a sample that was representative of national statistics in terms of sex, age, ethnicity or education, preventing full generalizability. Importantly, although more than half of our sample fell into the 25–34 years age group, we recruited participants that were 18 years or older which implies a very broad age range. Future studies could benefit from a more representative community sample, and the field could also benefit from the examination of these associations in youth or adolescent populations to foster a more nuanced understanding of these relations. Moreover, only a few individuals in the sample indicated having a current psychiatric diagnosis, and therefore could not be analyzed separately because of underpowered design. However, future studies are needed to look at the interplay between trajectories of psychopathology, ER and IU in clinical populations. Moreover, a limitation also concerns the longitudinal dropout in our sample across the timepoints, with the last timepoint showing a large dropout. Future studies should consider strategies to minimize longitudinal dropout and employ more rigorous sample selection techniques. Lastly, an important future avenue for investigation would be to examine the association of specific ER strategies, such as acceptance or rumination, with IU and CCF, and how they might influence psychopathology in the long run.

## Conclusion

In the present study, we identified ER difficulties to be an important pathway through which IU is associated with increased mental health problems during the pandemic. While both IU and ER difficulties seemingly serve as risk factors, our findings account for the role of CCF as a protective factor in acting as a safeguard for mental health in the face of uncertainty and global major stressors. Importantly, our findings practically indicate that it might be of utmost importance to provide access to trainings and programs that seek to enhance these protective factors when faced with a global crisis of a similar nature as the COVID-19 pandemic.

## Supplementary Information

Supplementary Table S1.

## Data Availability

The datasets used and/or analyzed in the current study will be made available without reservations from the corresponding author on reasonable request.
