# Supplementary material for: Interplay between uncertainty intolerance, emotion regulation, cognitive flexibility, and psychopathology during the COVID-19 pandemic: a multi-wave study
Source: Sci Rep. 2023 Jun 17;13:9854. doi: 10.1038/s41598-023-36211-3 (PMC10276821; doi:10.1038/s41598-023-36211-3)
Supplement: Supplementary file 1 — Supplementary Table S1. [file 41598_2023_36211_MOESM1_ESM.pdf]

Interplay between uncertainty intolerance, emotion regulation, cognitive flexibility, and psychopathology during the COVID-19 pandemic: A multi-wave study

Malvika Godara, Jonas Everaert, Alvaro Sanchez-Lopez, Jutta Joormann & Rudi De Raedt

Supplementary Table S1. Panel A provides descriptives of IU, ER difficulties, CCF, depression, anxiety and stress (mean and standard deviation) at each of the five timepoints. Panel B provides a correlation matrix indicating the associations between the slopes of IU, ER difficulties, CCF, depression, anxiety and stress.

A

Construct	Timepoint 1 Mean (*SD*)	Timepoint 2 Mean (*SD*)	Timepoint 3 Mean (*SD*)	Timepoint 4 Mean (*SD*)	Timepoint 5 Mean (*SD*)
**IU**	30.96 (10.34)	31.21 (13.80)	35.2 (14.48)	32.67 (13.71)	33.3 (12.92)
**ER difficulties**	18.39 (10.95)	18.62 (11.34)	19.93 (10.77)	19.13 (11.11)	21.83 (10.08)
**CCF**	69.81 (29.83)	72.29 (30.79)	65.11 (34.42)	66.38 (33.13)	64.93 (34.34)
**Depression**	6.99 (5.82)	7.42 (5.84)	7.02 (5.69)	7.49 (5.94)	7.51 (6.03)
**Anxiety**	5.95 (5.46)	5.93 (5.30)	6.21 (5.59)	6.34 (5.68)	6.65 (5.80)
**Stress**	7.13 (5.63)	7.20 (5.07)	7.59 (5.60)	7.25 (5.40)	7.74 (5.62)

B

**IUS**	1.00					
**ER difficulties**	.16*	1.00				
**CCF**	-.14*	-.26**	1.00			
**Depression**	.18*	.34***	-.12*	1.00		
**Anxiety**	.60***	.15*	-.14*	.18*	1.00	
**Stress**	.20**	.35***	-.15*	.19*	.24**	1.00
